# Pilot study with randomised control of dual site theta burst transcranial magnetic stimulation (TMS) for methamphetamine use disorder: a protocol for the TARTAN study

**DOI:** 10.1186/s40814-024-01498-0

**Published:** 2024-05-09

**Authors:** Tarun Yadav, Buddhima Lokuge, Melissa A. Jackson, Emma K. Austin, Paul B. Fitzgerald, Amanda L. Brown, Bryan Paton, Marcia Sequeira, Martin Nean, Llewllyn Mills, Adrian J. Dunlop

**Affiliations:** 1 Drug and Alcohol Clinical Services, Hunter New England Local Health District, Newcastle, Australia; 2 School of Medicine and Public Health, University of Newcastle, Callaghan, Australia; 3 School of Medicine and Psychology, College of Health & Medicine, Australian National University, Canberra, Australia; 4 Monarch Mental Health Group, Sydney, Australia; 5 School of Psychology, Hunter Medical Research Institute, University of Newcastle, Callaghan, Australia; 6 Discipline of Addiction Medicine, Central Clinical School, University of Sydney, Camperdown, Australia; 7 Drug and Alcohol Services, South Eastern Sydney Local Health District, Camperdown, Australia; 8 The Langton Centre, Surry Hills, Australia; 9 NSW Drug & Alcohol Clinical Research & Improvement Network, St Leonards, Australia

**Keywords:** Methamphetamine use disorder, Transcranial magnetic stimulation, Theta burst stimulation, Dorsolateral prefrontal cortex, Orbitofrontal cortex, Addiction

## Abstract

**Background:**

Transcranial magnetic stimulation (TMS) (including the theta burst stimulation (TBS) form of TMS used in this study) is a non-invasive means to stimulate nerve cells in superficial areas of the brain. In recent years, there has been a growth in the application of TMS to investigate the modulation of neural networks involved in substance use disorders. This study examines the feasibility of novel TMS protocols for the treatment of methamphetamine (MA) use disorder in an ambulatory drug and alcohol treatment setting.

**Methods:**

Thirty participants meeting the criteria for moderate to severe MA use disorder will be recruited in community drug and alcohol treatment settings and randomised to receive active TMS or sham (control) intervention. The treatment is intermittent TBS (iTBS) applied to the left dorsolateral prefrontal cortex (DLPFC), then continuous TBS (cTBS) to the left orbitofrontal cortex (OFC). Twelve sessions are administered over 4 weeks with opt-in weekly standardized cognitive behaviour therapy (CBT) counselling and a neuroimaging sub-study offered to participants. Primary outcomes are feasibility measures including recruitment, retention and acceptability of the intervention. Secondary outcomes include monitoring of safety and preliminary efficacy data including changes in substance use, cravings (cue reactivity) and cognition (response inhibition).

**Discussion:**

This study examines shorter TBS protocols of TMS for MA use disorder in real-world drug and alcohol outpatient settings where withdrawal and abstinence from MA, or other substances, are not eligibility requirements. TMS is a relatively affordable treatment and staff of ambulatory health settings can be trained to administer TMS. It is a potentially scalable and translatable treatment for existing drug and alcohol clinical settings. TMS has the potential to provide a much-needed adjuvant treatment to existing psychosocial interventions for MA use disorder. A limitation of this protocol is that the feasibility of follow-up is only examined at the end of treatment (4 weeks).

**Trial registration:**

Australia New Zealand Clinical Trial Registry ACTRN12622000762752. Registered on May 27, 2022, and retrospectively registered (first participant enrolled) on May 23, 2022, with protocol version 7 on February 24, 2023.

## Background

Amphetamine-type stimulants, including methamphetamine (MA), are the second most used illicit drug class. An estimated 29 million people worldwide used amphetamines in 2019 [1], with 7 million estimated to be dependent [2]. Poor health outcomes are seen particularly among people who use MA several times a week [3], including psychosis, depression, anxiety, blood-borne virus transmission, sexually transmitted infections and cardio/cerebral vascular events [4, 5]. MA use disorders are estimated to cost Australia around $AUD 3.2 billion a year [6].

The current standard of care for MA use disorder relies on psychosocial interventions (primarily cognitive behaviour therapy-based approaches) [7] with modest effectiveness. As with other substance use disorders, combination counselling with medications and/or newer treatment modalities such as neurostimulation may increase the effectiveness of treatment [3, 8]. There are currently several forms of neuromodulation, both invasive and non-invasive, which are being investigated to enhance the treatment of substance use disorders [8].

Transcranial magnetic stimulation (TMS) (including the theta burst stimulation (TBS) form of TMS used in this study) is a non-invasive means to stimulate nerve cells in superficial areas of the brain. It is a form of neuromodulation that induces a hyperpolarization (high frequencies) or depolarization (low frequencies) of neurones through electromagnetic induction. The repeated excitation or inhibition through this means of groups of neurones changes the activity of the specific regions of the brain targeted and potentially the strength of connections with subcortical areas of the brain where these neurones project [9]. The neuromodulatory and therapeutic effects of TMS therefore depend on the location, frequency and intensity of magnetic induction applied.

TMS has been used for mood disorders since the late 1980s and has been Therapeutic Goods Administration (TGA) approved as a treatment for major depressive disorder since 2007 in Australia. We now have over two decades of extensive behavioural, electrophysiological and neuroimaging work to describe the efficacy and safety of TMS with multiple review articles in this regard [10, 11].

In recent years, there has been an exponential growth in the application of TMS to investigate the modulation of neural networks involved in addiction including in alcohol, cocaine, opioid, cannabis and tobacco use disorders [12]. Additionally, the conceptual framework for designing TMS clinical studies and research protocols for alcohol and other drugs has been well described in a recent consensus paper published by over 50 scientists with expertise in this area [12].

Studies of TMS for substance use disorders (SUD) have targeted cortical areas of the brain involved in reward neurocircuitry. These regions include the prefrontal cortical network including the dorsolateral prefrontal cortex (DLPFC) and the orbitofrontal cortex (OFC) which have important functions in inhibitory control, a neurobehavioral output often impaired in patients with SUDs [13, 14]. Reduced inhibitory control and disinhibition are also associated with relapse susceptibility [15–19].

Furthermore, the DLPFC and surrounding networks are also associated with substance craving, and craving is a predictor of continued substance use and a major clinical feature of SUD associated with poor treatment outcomes and relapse [20–22]. In addition to TMS’s effects on brain reward neurocircuitry, TMS neuromodulation has proven beneficial in reducing symptoms of co-occurring psychiatric disorders/symptoms (e.g. depression) which may be further perpetuating and/or exacerbating an individual’s SUD [8].

Ekhtiari et al. strongly recommend that studies of TMS combine objective biological markers with self-reported outcome measures such as craving/cue reactivity. This is an additional means to understand and corroborate the changes involved in TMS interventions for SUD. Specifically, the impact of treatment on dysregulated cue-induced craving-related cognitive processes in SUDs. They recommend the addition of neuroimaging in SUD research involving TMS [12].

TMS has been demonstrated to have a low rate of treatment-emergent side effects or adverse events and is well tolerated [23]. Systematic reviews have also looked specifically at the safety of the TBS form of TMS used in this study and found few adverse events and low risk of serious adverse events consistent with conventional TMS [24–26].

### Using shorter TBS protocols: shorter and more time-efficient

This pilot study examines several novel TMS parameters in the treatment of MA use disorder that available evidence suggests may offer advantages over conventional TMS. These novel adaptations to existing TMS protocols are the use of TBS protocols of TMS that require significantly shorter durations of treatment (e.g. 2 min vs 30 min) and targeting two areas involved in brain reward neurocircuitry during sessions (the OFC and DLPFC).

TBS is a form of TMS where magnetic stimulation is applied in very short bursts (three pulses at a time) but at high frequency (usually around 50 Hz) and repeated at an inter-burst interval of 200 ms. The theoretical basis for TBS as a therapeutic modality is that modulating theta activity in turn alters gamma activity critical for neocortex-mediated cognitive functions [27].

Two types of TBS have been studied. Intermittent TBS (iTBS)—brief stimulation trains followed by a period of rest, e.g. 2 s of stimulation followed by 8 s of rest. Secondly, continuous TBS (cTBS)—brief stimulation trains continuously, e.g. packets of stimulation bursts are administered every 200 ms continuously for 20–40 s. Although there is variability in individual responses, the average effects of iTBS and cTBS on the brain are opposite. iTBS produces an increase in local cortical excitability while cTBS produces a decrease in excitability [27]. Some studies have suggested that both iTBS and cTBS induce changes in cortical activity that persist for longer periods of time than the effects produced by standard TMS protocols, despite taking only a fraction of the time to apply [27]. Additionally, the safety and efficacy of accelerated TMS/TBS protocols utilising multiple treatment sessions in a day have been demonstrated in studies including for treatment-resistant depression [28, 29].

While previous studies have looked at targeting individual sites (most commonly the DLPFC) in SUDs, this study will involve dual targeting of the DLPFC with (excitatory) iTBS, then the application of (inhibitory) cTBS to the OFC. Many of the TMS studies to date have applied TMS to the DLPFC in an effort to decrease craving [12, 30]. This area has an important role in executive and inhibitory control, often impaired in patients with SUDs, and disinhibition is also associated with relapse [12–16].

While upregulating the DLPFC has theoretical and empirical benefits in SUDs, from a craving and relapse perspective however, the majority of clinical neuroimaging studies demonstrate that the OFC and anterior cingulate cortex are regions that are more directly involved in craving, and resting functional connectivity among these regions is critical in relapse [31, 32]. Recently, Hanlon et al. have published results of several studies targeting the OFC with TMS/TBS applied to the left frontal pole to decrease connectivity in the circuit involving OFC [31, 33–35]. Decreasing connectivity, Hanlon et al. argue in this circuit through cTBS that it may reduce substance-induced pathological connectivity and ultimately dampen craving and improve clinical outcomes [33]. Hanlon et al. demonstrated that six sessions of cTBS targeting the OFC (location at Fp1) delivered in a single day is feasible and tolerable (six trains of FP cTBS) [33]. In addition to tolerability, they provide neuroimaging corroboration of their hypothesis that cTBS at this location led to attenuation of areas of the salience network (typically engaged by drug cues) in study participants who were cocaine- and alcohol-dependent. While these studies have not directly demonstrated the effect of cTBS on the attenuation of drug cue-induced craving, they are robust “proof of principle” of the value of cTBS at OFC in SUDs. Based on this data, we believe utilising these parameters in this pilot study is warranted given the potential synergistic benefits in terms of attenuation of craving.

While bilateral stimulation and the use of TBS are novel in MA use disorder, it has been used extensively in mental health. Studies and reviews over the last two decades of TMS safety in clinical settings have found it to be safe, with a rare risk of serious complications [36]. Recently, Zhao et al. examined the application of TBS to the dual sites of DLPFC and OFC for MA use disorder [37]. They found this TBS protocol efficacious; however, the context of this study was an inpatient treatment centre where patients were withdrawn from all substances prior to TBS. Our study will occur in an outpatient setting where withdrawal is not a pre-requisite. We found several approved trials registered on ClinicalTrials.gov underway in the USA utilising cTBS and targeting the OFC for treatment-seeking adults with cocaine and alcohol use disorders [38]. An additional registered RCT will utilise simultaneous iTBS for DLPFC and cTBS for OFC (also called mPFC) as in our proposed study.

### Trial objectives and endpoints

The primary objectives are as follows:

- Assess the feasibility and preliminary safety of using TMS for the treatment of moderate to severe MA use disorder in outpatient drug and alcohol settings.

The secondary objectives are as follows:

- Examine treatment adherence rates of participants for the use of TMS and counselling for MA use disorder
- Assess the impact of TMS on substance use in patients with MA use disorder
- Assess the impact of TMS on cravings in patients with MA use disorder
- Assess the impact of TMS on cognition in patients with MA use disorder
- Assess patient experiences with the use of TMS for MA use disorder

N.b. The study is not powered for testing differences between arms. Reporting the impact of TMS on substance use, cravings and cognition will be descriptive and exploratory in nature.

### Study design

This is a pilot study with double-blinded randomised controls. The study population are treatment-seeking adults with moderate to severe MA use disorder attending Hunter New England Local Health District (HNELHD) sites and associated community organisations.

#### Recruitment and retention

The recruitment strategy for this study will incorporate approved advertisements being displayed in participating waiting rooms and other suitable locations, including community-based settings such as doctor’s surgeries and drug and alcohol treatment-related non-government organisations (NGOs). Up to 30 participants will be recruited for the main study. Consent will be obtained by study researchers and including consent to participate in the imaging sub-study which is limited to 20 participants due to budgetary reasons. Details of the sub-study including a detailed protocol are available on request from the corresponding authors. Each participant will be involved in the study for the 4-week treatment period. We anticipate it will take 30 weeks to recruit this cohort. Participants receive reimbursements of retail gift vouchers to cover time and expenses associated with study participation. Treatment adherence and follow-up will be actively pursued through measures including SMS text reminders and calls.

#### Trial schema


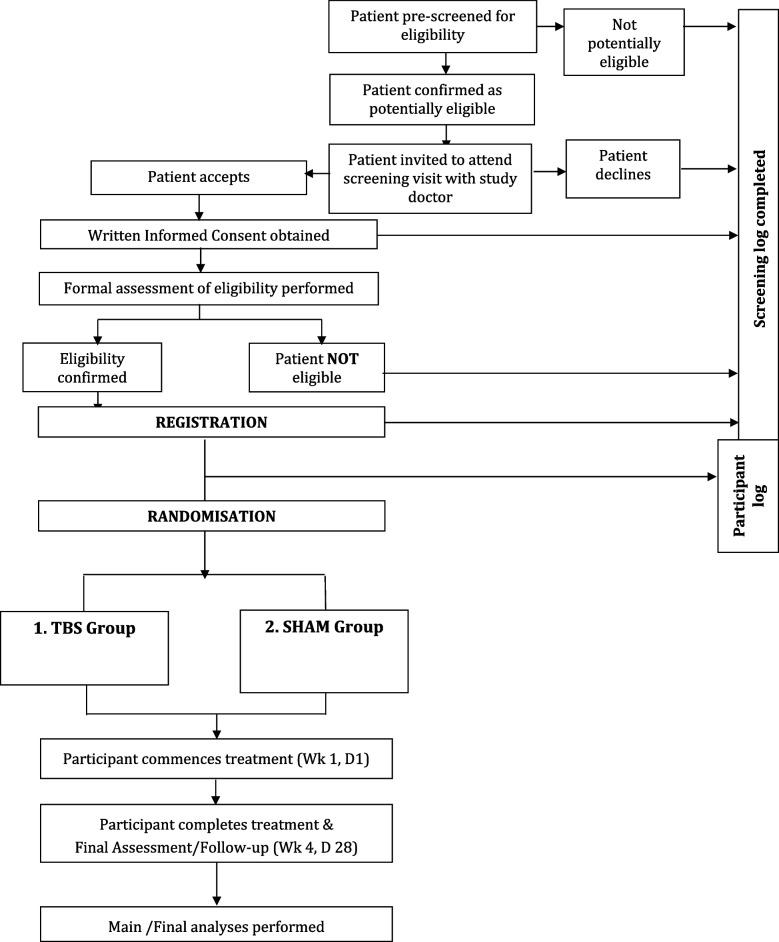


#### Participant eligibility

Inclusion criteria

- Age between 18 and 65 years
- Meet the Diagnostic and Statistical Manual of Mental Disorders (DSM 5) criteria for moderate to severe MA use disorder
- Self-report current MA use (one day plus) in the past month
- Able to give written informed consent
- Willing and able to comply with the requirements of the study

The study inclusion criteria are deliberately broad, and abstinence or withdrawal from methamphetamine are not eligibility requirements.

Exclusion criteria

- Psychoactive substance use requiring withdrawal management pharmacotherapy in the 28 days preceding eligibility screening (e.g. alcohol withdrawal requiring diazepam) excluding nicotine replacement therapy
- Current pharmacotherapy for amphetamine use disorder (e.g. dexamphetamine) in the 28 days preceding eligibility screening
- Current diagnosis of bipolar disorder, schizoaffective disorder and schizophrenia that is deemed by research team psychiatrists not to have been drug-induced. Psychotic disorder not associated with drug use per DSM 5 criteria. Psychosis not otherwise specified (NOS), in remission, or drug-induced psychotic episodes are not exclusion criteria since these may be related to methamphetamine misuse
- Severe uncontrolled medical condition, diagnosis of neurological disorder or neurocognitive disorder; prior neurosurgical procedure
- Contraindications to TMS (e.g. patients with epilepsy or seizure disorder, patients with implanted ferromagnetic equipment in their face or skull near the stimulation target, Cochlear implants, metal implant or electronic devices in the head, brain aneurysm clips/coils, VNS, pacemakers, deep brain stimulation, VP shunts, brain or neck stents, epilepsy, medical pump, hearing disorder, recent head injury)
- Current or planned pregnancy
- History of ECT treatment within the past 3 months
- History of any previous TMS treatment (to ensure the sham (placebo) arm is not unblinded due to familiarity with TMS)
- Additional functional MRI-related exclusions apply to participation in the imaging sub-study including those with any implanted devices (e.g. pacemaker, coronary stent, defibrillator or neurostimulation device or any other implanted device or metal in body) and participants with severe claustrophobia

### The intervention

TMS/TBS will be administered with a TGA-registered TMS device Neuro-MSD, Fig8 Coil. A treatment and sham protocol will be created in Neuro-MS.NET software to have consistency of delivery without compromising the blinding. All research team members involved in providing the intervention will receive certified training and accreditation by experienced trainers from Neurocare groups or other suitably qualified training organisations if necessary.

Prior to the commencement of TBS treatment, single-pulse TMS will be used to measure the resting motor thresholds (RMT) for the abductor pollicis brevis (APB) in the right hand in all subjects using standard published methods [39]. The RMT is used to determine the intensity of the TBS treatment for each individual participant.

Scalp locations at which the TMS coil will be placed will be determined using standard EEG 10–20 system landmarks. Two locations will be targeted. iTBS is applied to the left DLPFC, localised at the F3 EEG site. Then, cTBS is applied to the left OFC, localised at the FP1 EEG site. These are localised using a computerised measurement tool based on head measurements (http://clinicalresearcher.org/software.htm). This is a commonly used approach to overcome limitations with standard methods for locating treatment sites which do not factor for individual variations in head size.

The active TBS protocol is sequenced in a session as follows. iTBS will be delivered at the left DLPFC (F3) as 3-pulse 50-Hz bursts applied at 5 Hz (i.e. 50 Hz burst of 3 pulses delivered every 200 ms) with a 2-s train of TBS repeated every 10 s (i.e. 2 s of TBS followed by a 8-s rest). Total number of pulses is 600. Each TBS treatment session will take approximately 3 min. During the initial treatment sessions, the amplifier output will be escalated (over 30 s) from 70 to 110% RMT to enhance tolerability. For clients who experience discomfort and are not able to comfortably tolerate increases in intensity, the level will be maintained at the maximally tolerable level.

Following this, cTBS will be delivered at the left OFC: One train of cTBS will be applied over the left frontal pole (Fp1) (3 pulses of 50-Hz bursts applied at 5 Hz, 15 pulses/s, 600 pulses/train; 100% RMT). During the cTBS procedure, the amplifier output will be escalated during the first train (over 30 s) from 70 to 100% RMT to enhance tolerability. For clients who experience discomfort and are not able to comfortably tolerate this intensity, the level will progressively be decreased until a comfortable intensity is reached. For both iTBS and cTBS, participants will be withdrawn if they are unable to tolerate treatment at 70% of the RMT at the end of the third day of treatment.

Three sessions of iTBS followed by cTBS will be administered with a 10-min break between the first, second and third sessions. This interval and frequency were determined based on studies showing accelerated multiple iTBS cycles delivered with an interval between each within a day as safe and efficacious [28, 29]. This was also supported by the expert opinion of study investigators with extensive TMS research experience. Treatment will be provided on 3 days of the week for a total of 4 weeks (12 sessions or days of treatment in 28 days).

#### Control and blinding

In TMS trials, it involves controls utilising sham devices to mimic the sight, sound and feel of real stimulation, while avoiding any direct stimulation of the central nervous system [12]. For this study, we will utilise the integrated sham stimulation system in the Neuro-MS.NET software which allows sham stimulation to be delivered by electrodes that create a proportional sensory stimulation to the real TMS intervention. The electrodes will be placed on the exact same location near the coil for both groups, though they will only be active during the sham stimulation, not TBS sessions. There is also synchronous sound masking (created by a speaker located near the discharging coil). The synchronous electrical stimulation is given through electrodes fixed on the head along the front edge of the coil to mimic the sensation. The electrical stimulation amplitude for TMS-naïve subjects was from 2 to 5 mA, providing superficial skin sensations but not stimulation of the brain stimulation. This software has been validated by Neurosoft as providing effective blinding for TMS-naïve subjects and, to a lesser extent, TMS experience participants. For this study, we will exclude participants who have experience using TMS. The study will be double-blinded, i.e. doctors, nurses and researchers involved in the study will be blinded.

Participants will be randomised in a 1:1 ratio between active TMS and sham. A computer-generated randomisation schedule in the TMS device will randomise participants to active and sham interventions. The allocation will not be accessible by researchers delivering the intervention but will be available at the end of the study to independent statisticians performing analysis of the data.

#### Allocation concealment

This will be ensured by applying rules and user rights within the TMS device software. The randomisation allocation will not be able to be generated until after the patient recruitment has been completed. The statistician coordinating data analysis only will have access to the Neuro-MS treatment allocation settings for each participant. At the end of the study treatments, we will ask researchers delivering the sessions, and participants will be asked to nominate if they believe they received TBS or sham sessions.

#### Counselling

All participants will be offered a standard of care for MA use disorders, involving psychosocial counselling for the duration of the study and referral to appropriate services post-study as clinically indicated. Importantly, study-related counselling sessions will provide support to participants through psychoeducation into the neurocircuitry of MA use disorder, relapse prevention for MA dependence and TMS treatment adherence. Counselling sessions will be offered face to face or by telehealth or telephone as requested. Clients will be offered one counselling session per week during the 4 weeks of treatment. Counselling will be performed by a trained HNELHD Drug and Alcohol Clinical Services counsellor independent of the study.

### Outcome measures

Feasibility will be assessed by recording the number of study referrals, the proportion of those who are deemed eligible and consented and their attendance and completion of scheduled treatments. A semi-structured questionnaire of patient experience will be administered post-treatment. Safety is monitored by the recording of all adverse events.

The preliminary efficacy of TMS on substance use will be determined by changes in self-reported substance use, collected using the Timeline Follow Back for MA use (a validated tool used to recall the previous 28 days of substance use) and the Australian Treatment Outcome Profile (a 28-item validated tool for drug and alcohol outcome measures including substance use, and social and wellbeing indicators). Self-report data will be verified by urine drug screens collected at baseline, mid-treatment and end of treatment.

The impact of TMS on specified cognitive domains is assessed by two cognitive tests done at baseline and end of treatment. These are the Go/No-Go test examining response inhibition (inhibitory control), and a cue reactivity paradigm (assessing reactivity of craving measured with a visual analogue scale, to drug and non-drug cues using a validated MA and neutral image database).

### *Study schedule (see *Table 1* for details)*

**Table 1:** Timeline of assessments

Assessment/form	**Screening**		**Treatment (weeks 1–4)**				
	Pre-screen	Screen	Baseline	Days 2–28			
			Week 1^a^ (D1)	Week 2^a^ (days 8–14)	Week 3^a^ (days 15–21)	Week 4^a^ (days 22–27)	Final assessment (day 28) within 7 days of last treatment
Pre-screening	*√*						
Eligibility, consent & registration		*√*					
Current MA/substance use & MA/substance use history add treatment history		√					
Medical & mental health history (incl. contraindications) and examination (medical and mental health)		*√*					
General health assessment							*√*
Concomitant medications		*√*	*√*				
Medical protocol: RMT calculation			*√*				
Socio-demographics, participant education & treatment adherence			*√*				
TMS—treatment safety screening assessments			*3*	*3*	*3*	*3*	
Study counselling (both arms)			√	√	√	√	
Vital signs			√	√	√	√	√
Adverse event log			*√*	*√*	*√*	√	√
Urine sample (for UDS)		*√*	*√*		*√*		√
Pregnancy test (where applicable)		*√*					
Timeline Follow Back (MA use only)			*√*	*√*	*√*	√	√
MA craving visual analog scale (VAS)			*√*	*√*	*√*	√	√
MA cue-exposure paradigm & craving			*√*				√
Australian Treatment Outcome Profile (ATOP)			*√*				√
Mental health (DASS-21)			*√*				√
Cognitive testing (Go/No-Go)			*√*				√
Treatment experience							√

^a^These assessments are done at the first session of the week

The study doctors will establish RMT and set the TMS parameters for each participant and conduct the first treatment session as per the participant’s randomly assigned treatment. The study doctor (or delegate certified in TMS) will provide up to three sessions per week, as per the participant’s randomly assigned treatment. The study doctor will review the patient at the conclusion of the study treatments. Final study assessments will be conducted at the end of treatment. A participant will be defined as having completed the study once they have completed their week 4 post-treatment follow-up assessment or if they are prematurely withdrawn from the entire study (i.e. treatment and research visits).

Task-based functional magnetic resonance imaging (fMRI) will be done at baseline and end of treatment for those who opt-in to a neuroimaging sub-study. Scanning is conducted while cue reactivity and the Go/No-go tasks are undertaken. Simultaneous physiological monitoring (e.g. heart rate in and out of the scanner) will also be performed. Brain areas of interest include those involved in the reward, craving and cognitive control associated pathways, i.e. the cortico-limbic striatal systems. The neuroimaging procedures and assessments are contained in a sub-study protocol that will be published separately.

Participants will be advised at the time of consenting that the study treatment and counselling will not be available after the study period (end of week 4 of treatment). Participants requesting a referral for the continuation of psychosocial counselling will be provided referral options.

Participants who withdraw from the TMS component of the study will be invited to continue completing the research component of the study. Participants may be withdrawn involuntarily by the investigator (or delegate) if they meet the following criteria: participant experiences a severe or serious adverse event, thought to be related to the study device, which is not resolving, or they miss more than 6 (of 12) study TMS sessions. Participants have the option to stop treatment or revoke their consent at any time without giving a reason and no further information would be collected from the participant for the purpose of the trial.

### Termination

The Principal Investigator or Trial Management Committee may recommend stopping the trial should the number and/or severity of adverse events justify discontinuation of the study.

## Study assessments

### Safety

#### Safety event reporting

Information will be collected on standard adverse events and serious adverse events (SAE) with relevant data reported to the ethics committee and regulatory authorities as required by the study protocol and ethics approval. The study management committee will review interim safety data at regular intervals.

### Side effects of TMS/TBS [25, 40]

#### Common side effects

Local pain, discomfort, headache and neck pain—all generally mild, discontinuation rates due to these symptoms are low. Pain is thought to be due to stimulation of superficial nerves or facial muscles, neck pain related to uncomfortable positioning during treatment, and headache may relate to local scalp stimulation [40].

#### Severe side effects

The risk of seizures is very low with the incidence thought to be equivalent to the incidence of spontaneous seizures with antidepressant therapy (0.1–0.6%) [25, 40]. The risk is increased with high-frequency treatment and more intense treatment protocols; pre-existing neurological conditions, adolescent patients, substance use and concurrent medication changes may all impact the seizure threshold. The risk of TMS-induced hearing impairment is also very low [25]. Studies have found that when adequate hearing protection is used, no change in hearing after a course of TMS is seen. We mitigate this risk by the use of approved hearing protection (earplugs); prompt referral for auditory assessment of all individuals who complain of hearing loss, tinnitus, or aural fullness following completion of TMS; and excluding those with pre-existing hearing disorders from the study.

The study will utilise the safety guidelines published in the Australian Psychiatry “transcranial magnetic stimulation (TMS) safety: a practical guide for psychiatrists” [40]. In the unlikely event that a participant has a seizure during a treatment session, a standard protocol for managing seizures in outpatient clinic settings will be utilised.

#### Data management and analysis

All data collected for the study will be confidentially managed and stored using Research Electronic Data Capture (REDCap) data capture tools [41, 42] (refs). REDCap is a secure, web-based application designed specifically for research studies, hosted by Hunter New England Local Health District. The study investigators and study team will have access to the study data. The study management team will oversee data monitoring.

As a pilot study, data will be summarised by descriptive statistics for primary and secondary outcome measures. Outcomes will be summarised across the intervention and control arms of this study.

#### Primary outcomes

Feasibility will be assessed via reporting the number of study referrals, the proportion deemed eligible and consenting (*N* (%)). Safety will be assessed by reporting the number of adverse effects (*N*).

#### Secondary outcomes

Adherence will be reported as the frequency of eligible treatments completed per week (mean (standard deviation)/median (inter-quartile range)) by the treatment group. The potential impact of TMS on substance use will be assessed by reporting the self-reported use by week (mean (SD)/median (IQR)) for each treatment group, and the frequency of positive urine drug screens by week for each treatment group (*N* (%)). The potential impact of TMS on cravings will be assessed by reporting craving scores (mean (SD)/median (IQR)) for each treatment group. The impact of TMS on cognition will be assessed by reporting the Go/NoGo test commission error rate (mean (SD)/median (IQR)) for each treatment group. Patient experience will be assessed by reporting the frequency of Likert scale responses (*N* (%)) in a semi-structured questionnaire of patient experience.

#### Sample size

A pragmatic sample size of 30 (15 per arm) was chosen as the target sample size, due to the fact that this is the maximum number that we anticipate could be recruited in this population in the study time frame given the available resources (time, funding and staff). Feasibility will be reported as the proportion adhering to treatment per arm; however, no formal statistical comparisons between the arms will be performed as the study is not powered to do so. This sample will provide preliminary information regarding drop-out rates, adherence, completions etc., and preliminary data regarding efficacy, to inform a future larger, adequately powered study.

## Discussion

The study will examine the real-world feasibility of TBS in an Australian clinical setting supporting clients with MA use disorder. We will assess recruitment, treatment adherence, tolerability and patient acceptability of this novel treatment in the substance use disorder field. We will also examine preliminary efficacy although the study is not powered to assess this. The study will also provide novel information on neurobiological changes that occur during TMS treatment. Results will be used to inform the development of larger studies including an RCT examining TBS use for MA use disorder, potentially the first to do so internationally. DACS HNELHD is a member of the NSW Health-supported Drug and Alcohol Clinical Research and Improvement Network (DACRIN) group that would facilitate the translation of the results of this feasibility study into an RCT across a number of LHDs.

The potential for beneficial results among populations dependent on MA is significant. Currently, there are limited treatments available for MA use disorder, and those available show a limited efficacy in moderate and severe MA use disorder. TMS may enhance the effectiveness of existing treatment modalities. The TMS protocols examined in this study have the advantage of being less demanding in terms of treatment duration for clients and resourcing for Drug and Alcohol Clinical Services.

## Data Availability

Study data are available from the corresponding author on reasonable request.

## Abbreviations

ACC: Anterior cingulate cortex
AE: Adverse event
APB: Abductor pollicis brevis
ATOP: Australian Treatment Outcome Profile
CBT: Cognitive behaviour therapy
CNS: Central nervous system
DASS-21: Depression Anxiety Stress Scales
DACS: Drug and Alcohol Clinical Services
DLPFC: Dorsolateral prefrontal cortex
DSM: Diagnostic and Statistical Manual of Mental Disorders
EEG: Electroencephalogram
HREC: Human Research Ethics Committee
HNELHD: Hunter New England Local Health District
MA: Methamphetamine
MRI: Magnetic resonance imaging
NGO: Non-government organisation
OFC: Orbitofontal cortex
RCT: Randomised controlled trial
RMT: Resting motor threshold
SUD/s: Substance use disorder/s
TBS: Theta burst transcranial magnetic stimulation
iTBS: Intermittent theta burst transcranial magnetic stimulation
cTBS: Continuous theta burst transcranial magnetic stimulation
TLFB: Timeline Follow Back
TGA: Therapeutic Goods Administration
TMS: Transcranial magnetic stimulation
UDS: Urine drug screen
VAS: Visual analog scale
